# Identification and analysis of a unique group of glycoside hydrolase family 188 sequences with an altered sulfonate binding residue

**DOI:** 10.17912/micropub.biology.001303

**Published:** 2024-09-25

**Authors:** Anne E. Backlund, Melanie A. Higgins

**Affiliations:** 1 Biological Sciences, University of Alabama, Tuscaloosa, Alabama, United States

## Abstract

Sulfoquinovosyldiacylglycerol (SQDG) is a plant sulfolipid that plays a major role in the global sulfur cycle. Bacteria contain sulfoglycolytic pathways that are responsible for metabolizing SQDG which requires initial delipidation by a sulfolipase and sulfoquinovosidase (SQase). Recently, a new group of SQases was discovered and have been categorized in a separate glycoside hydrolase family (GH188). Here we have identified a subset of GH188s with an altered sulfonate binding residue. We found that these GH188s have a distinct dimer interface and are found in unique gene clusters that may represent new sulfoglycolytic pathways. Further investigation into these enzymes could broaden our understanding of this new glycoside hydrolase family and uncover diverse sulfoglycolytic pathways.

**Figure 1. GH188 analysis:**
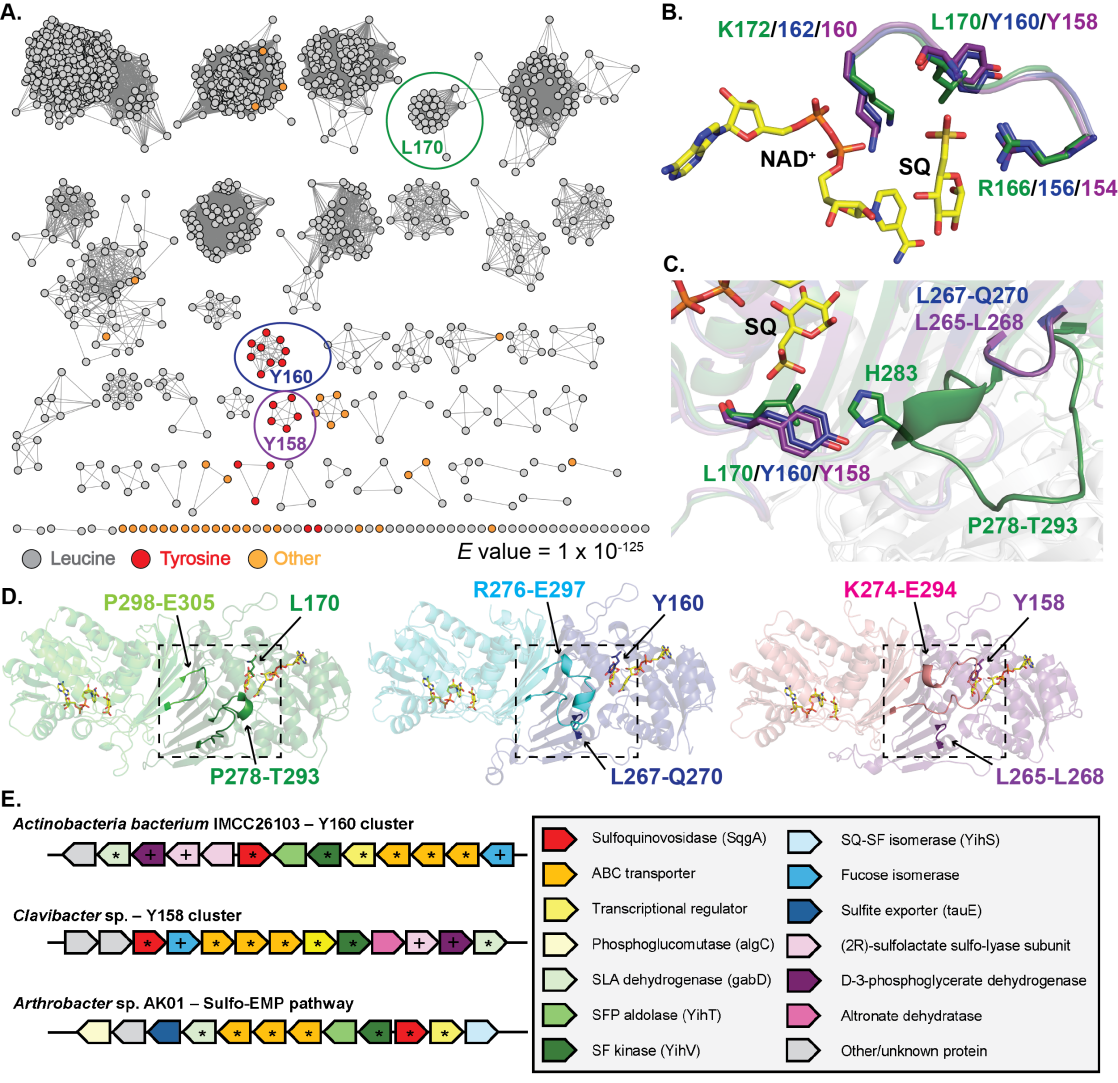
**A** ) SSN for GH188 sequences. The corresponding residue at position L170 with respect to characterized GH188 sequence are differentially colored. The SSN cluster containing the structurally characterized GH188 is circled in green, the Y160 SSN cluster in blue, and Y158 cluster in purple. **B** ) Comparison of sulfonate binding site residues for the GH188 crystal structure (green; PDB ID 8QC2) (Kaur et al., 2023) and the AlphaFold models for representative Y160 (blue) and Y158 (purple) sequences. NAD ^+^ and SQ are shown in yellow and are from the GH188 crystal structure. **C** ) Comparison of the loop region surrounding the L170, Y160, and Y158 side chain and **D** ) the dimer interface involving that loop region. Nitrogens are shown in blue, oxygens in red, sulfur is dark yellow, and phosphorus in orange. **E** ) Genomic neighborhood surrounding GH188 sequences that correspond with Y160 and Y158, and the Sulfo-EMP pathway (Kaur et al., 2023). Plus signs represent conserved genes between Y160 and Y158 gene clusters and stars represent conserved genes between Y160, Y158 and the Sulfo-EMP pathway gene clusters.

## Description

Introduction :

Sulfoquinovose (6-deoxy-6-sulfoglucose), also known as SQ, is the head group of a plant sulfolipid, sulfoquinovosyldiacylglycerol (SQDG), which comprises plant and algal thylakoid membranes (Shimojima, 2011) . SQ is estimated to be biosynthesized and degraded at 10 billion tons annually, so it is a major player in the global sulfur cycle (Harwood & Nicholls, 1979) . Many bacteria harbor sulfolytic pathways to release sulfite and glucose from SQ which can be used as a source of sulfur and carbon, respectively. However, this requires initial delipidation of SQDG by the sequential action of a sulfolipase and sulfoquinovosidase or SQase.

The first known SQases comprise a subset of the glycoside hydrolase family 31 (GH31) enzymes which utilize a retaining a mechanism to hydrolyze a 6-sulfo-α-D-quinovosyl diacylglycerol into 6-sulfo-α-D-quinovose and a 1,2-diacylglycerol (Shibuya & Benson, 1961; Speciale et al., 2016) . Recently, a new family of SQases was discovered after recognizing that some SQ catabolic gene clusters did not appear to contain a classical GH31 SQase (Kaur et al., 2023) . This new family, glycoside hydrolase family 188 (GH188), consists of >1000 sequences and are found in bacteria, plants, and archaea. GH188 enzymes use an atypical oxidoreductive mechanism with NAD ^+^ . Structural analysis revealed that GH188 enzymes have conserved catalytic residues, along with a unique sulfonate binding site. Here, we show that the sulfonate binding site is conserved amongst almost all members of GH188. However, we have identified two groups of GH188 enzymes with one residue variation in the sulfonate binding site that may result in rearrangement of loop regions and an altered dimer interface. These enzymes are also found in unique gene clusters that could represent new sulfoglycolytic pathways.

GH188 sequence similarity :

GH188 structures in complex with NAD ^+^ and SQ reveal a unique sulfonate binding site that consists of interactions between the sulfonate oxygens and the side chain nitrogens of R166, side chain nitrogen of K172, and amino nitrogen of L170 from GH188 (Kaur et al., 2023) . To see if these are fully conserved amongst the GH188 family, we performed multiple sequence alignment on all GH188 sequences previously generated (Kaur et al., 2023) . We found that R166 was found in 98.3% of sequences while L170 was found in 96.0% and K172 in 96.7% of sequences. We then mapped the residues at these positions to a Sequence Similarity Network (SSN) that we generated using the Enzyme Function Initiative Enzyme Similarity Tool (EFI-EST) (Oberg et al., 2023; Zallot et al., 2019) ( **Figure 1A** ). The alternative residues for R166 and K172 appeared to be found in random SSN clusters. However, we observed two separate SSN clusters that had a Tyr residue in place of Leu at position L170, one cluster with Y160 and one with Y158. There were also some isolated sequences scattered across the SSN that had a residue that differed from L170, Y160, and Y158. We chose to further investigate these two clusters’ structural features and genomic neighborhoods.

Structural features :

To see if there is a change in the sulfonate binding site for the Tyr variants, we obtained AlphaFold models of the GH188s from *Actinobacteria bacterium* IMCC26103 (Uniprot ID A0A7D6IM98) which is from the Y160 cluster and *Clavibacter* sp. (Uniprot ID A0A388P5E9) from the Y158 cluster. When comparing these AlphaFold models to a GH188 crystal structure in complex with NAD ^+^ and SQ (PDB ID 8QC2) (Kaur et al., 2023) , we see that the sulfonate binding sites remain intact ( **Figure 1B** ). This was not surprising, since the interaction with the sulfonate is with the amino nitrogen of Leu or Tyr. We then looked at the side chain interactions, to see if there were any differences between the GH188 crystal structure and the Tyr variant models. For the GH188 crystal structure, H283 from loop P278-T293 is in close proximity to L170 ( **Figure 1C** ). However, this loop is truncated in both Tyr variant models. Interestingly, when we modeled the Tyr variants as dimers similar to the GH188 crystal structure, we found that these variant models contain an extended loop from the adjacent monomer that may be able to replace the interactions missing due to the truncated loop ( **Figure 1D** ). This results in an altered dimer interface. Further analysis will be required to validate the Tyr variant models and determine if these changes in the sulfonate binding residue and dimer interface affect or abolish activity for this subset of GH188 enzymes.

Genomic Neighborhood:

Lastly, we wanted to see if the Tyr variants were found in gene clusters dedicated to degrading SQDG. The gene clusters were similar within each Y160 and Y158 SSN cluster, so we chose one representative gene cluster from each to analyze. The gene cluster from *Actinobacteria bacterium* IMCC26103 represents the Y160 SSN cluster, and the gene cluster from *Clavibacter* sp. represents the Y158 SSN cluster **( Figure 1E )** . The Y160 variant gene cluster contains an SLA dehydrogenase, D-3-phosphoglycerate dehydrogenase, an alpha and beta (2R)-sulfolactate sulfo-lyase subunit, SFP aldolase, SF kinase, a transcriptional regulator, ABC transporters, and fucose isomerase. The Y158 variant gene cluster is mostly conserved with the Y160 cluster, with the exception that it only has one (2R)-sulfolactate sulfo-lyase subunit and it does not contain the SFP aldolase. In addition, the Y158 gene cluster has an altronate dehydratase gene which is not present in the Y160 cluster.

We next wanted to compare these genomic neighborhoods with those of the five known sulfoglycolytic pathways that bacteria utilize to catabolize SQDG (Snow et al., 2021) . These are known as the sulfoglycolytic Embden-Meyerhof-Parnas (sulfo-EMP) pathway, the sulfoglycolytic Entner-Doudoroff (sulfo-ED) pathway, the sulfoglycolytic sulfofructose transaldolase (sulfo-SFT) pathway, the sulfoglycolytic transketolase (sulfo-TK) pathway, and the sulfoglycolytic SQ monooxygenase (sulfo-SMO) pathway. Although the Tyr variant gene clusters were not identical to any of the known sulfoglycolytic pathways, they most closely resembled the Sulfo-EMP gene cluster. When comparing the Tyr variant gene clusters with the GH188 gene cluster from *Arthrobacter* sp. AK01, which is from the Sulfo-EMP group, we found that all three clusters shared several genes, including an ABC transporter, a transcriptional regulator, sulfolactate dehydrogenase, and sulfofructose kinase. ( **Figure 1E** ). The Sulfo-EMP gene cluster and the Y160 variant gene cluster both contain sulfofructosephosphate aldolase, but this is not present in the Y158 variant gene cluster. Additionally, both Tyr variant gene clusters share some genes that are not present in *Arthrobacter* , including a fucose isomerase, (2R)-sulfolactate sulfo-lyase subunit, D-3-phosphoglycerate dehydrogenase. Since the Tyr variant gene clusters have some similarities and differences in their potential SQ metabolism genes when compared to know sulfolytic gene clusters, they could represent new sulfoglycolytic pathways.

Conclusion :

GH188 enzymes are a recently discovered glycoside hydrolase family with SQase activity and are involved in SQDG metabolism (Kaur et al., 2023) . Here we have identified a subset of GH188 sequences with an altered sulfonate binding residue and distinct dimer interface due to loop rearrangements. In addition, these GH188 sequences are found in unique gene clusters that may represent new sulfoglycolytic pathways. Altogether, these results further highlight the diversity of SQDG metabolism and lays the groundwork to investigate unique characteristics found in certain sequences from a newly discovered GH188 family of enzymes.
